# Avian influenza outbreaks in domestic cats: another reason to consider slaughter-free cell-cultured poultry?

**DOI:** 10.3389/fmicb.2023.1283361

**Published:** 2023-12-15

**Authors:** Piotr Rzymski

**Affiliations:** Department of Environmental Medicine, Poznan University of Medical Sciences, Poznan, Poland

**Keywords:** avian influenza, A/H5N1, zoonotic diseases, poultry industry, cultured meat, food safety, *Felis catus*

## Abstract

Avian influenza causes substantial economic loss in the poultry industry and potentially threatens human health. Over recent years, the highly pathogenic avian influenza A/H5N1 virus has led to devastating losses in poultry flocks and wild birds. At the same time, the number of mammalian species identified to be infected with A/H5N1 is increasing, with recent outbreaks in domestic cats, including household individuals, evidenced in July 2023 in Poland, ultimately creating opportunities for the virus to adapt better to mammalian hosts, including humans. Overall, between 2003 and 2023, over 10 outbreaks in felids have been documented globally, and in six of them, feed based on raw chicken was suspected as a potential source of A/H5N1, fuelling a debate on threats posed by A/H5N1 and methods to decrease the associated risks. This article debates that technology allowing the production of slaughter-free meat, including poultry, from cell and tissue cultures could be considered as a part of a mitigation strategy to decrease the overall burden and threat of adaptation of avian influenza viruses to human hosts. By shifting poultry production to the cultured meat industry, the frequency of A/H5N1 outbreaks in farmed birds may be decreased, leading to a reduced risk of virus acquisition by wild and domesticated mammals that have direct contact with birds or eat raw poultry and have close contact with human (including domestic cats), ultimately minimizing the potential of A/H5N1 to adapt better to mammalian host, including humans. This adds to the list of other benefits of cultured meat that are also reviewed in this paper, including decreased antibiotic use, risk of microbial contamination and parasite transmission, and environmental and ethical advantages over conventional slaughtered meat. In conclusion, further development and implementation of this technology, also in the context of poultry production, is strongly advocated. Although cultured poultry is unlikely to replace the conventional process in the near future due to challenges with scaling up the production and meeting the continuously increased demand for poultry meat, it may still decrease the pressures and threats related to the transmission of highly pathogenic avian influenza in selected world regions.

## 1 Introduction

In July 2023, the United States Department of Agriculture approved chicken meat made by two companies using animal cell culture technology for human consumption (The Good Food Institute, 2023). This decision followed an earlier pre-marketing assessment by the United States Food and Drug Administration, which included an evaluation of the production process and quality of the final product (FDA, 2022, 2023). Cultured meat (or cultivated meat, clean meat, slaughter-free meat, *in vitro* meat, lab-grown meat, or cellular agriculture) refers to the product obtained using emerging technologies that integrate the laboratory methods of *in vitro* cell culture with tissue engineering to produce animal muscle under a controlled environment (Choi et al., 2021). The starting point of this process is a collection of tissue through animal biopsy, isolation of stem cells (myosatellites and others, depending on the product, e.g., embryonic stem cells, adipose-derived stem cells, fibroblasts, and endothelial stem cells), their proliferation and differentiation, followed by tissue structuration using different techniques, depending on the type of produced meat (Post, 2012; Bodiou et al., 2020; Kang et al., 2021; Reiss et al., 2021; Handral et al., 2022).

Historically, the idea of cultured meat dates back to 1931 when Winston Churchill published an essay entitled “Fifty Years Hence” in Strand Magazine, republished in 1932 by Popular Mechanics, in which he envisioned the future and stated: “We shall escape the absurdity of growing a whole chicken in order to eat the breast or wing, by growing these parts separately under a suitable medium” (Churchill, 1931). However, it took eight decades to obtain the first meat for human consumption, a beef burger, using an *in vitro* cell culture technique in 2013. Since then, the technology has received substantial interest from the scientific community, private investors, as well as some national stakeholders, e.g., in China, which included the development of cultured meat in their recent 5-years agriculture plan covering the period of 2021–2025 (FAO, 2022). The first regulatory agency to approve cultured meat, i.e., cell-cultured chicken, was the Singapore Food Agency in December 2020 (Singapore Food Agency, 2020). However, considering that Singapore is not a major market for chicken (USDA, 2023b), this decision could be regarded as symbolic and important from a marketing point of view. On the contrary, the authorization of similar products in the United States represents a historical milestone in the food industry if the U.S. market is the largest chicken production globally, with approximately 21 million metric tons of broiler meat obtained in 2022 alone (USDA, 2023b). By the end of 2023, cultured meat is not authorized for consumption in the European Union, but it is known that the safety and quality of products obtained using such technology will be evaluated by the European Food Safety Authority acting upon Regulation 2015/2283 of the European Parliament and of the Council of 25 November 2015 on novel foods (European Food Safety Authority, European Centre for Disease Prevention and Control, European Union Reference Laboratory for Avian Influenza et al., 2023).

In the present paper, cultured meat is discussed in the context of the benefits of its introduction, with a particular focus on the potential mitigation of emerging threats posed by avian influenza viruses. The increasing number of A/H5N1 transmissions to different mammalian species increases the risk of better viral adaptation to a human host (Imai et al., 2013; Leguia et al., 2023; Venkatesan, 2023; WHO, 2023c). A recent outbreak of A/H5N1 infections in domestic cats, documented in 2023 in Poland (ECDC, 2023a), represents another reminder of these risks, mitigation of which justifies consideration of multifaceted measures, which also includes changes to conventional poultry production that are offered by the cultured meat technology. As indicated, cultured meat, in addition to its ethical and environmental advantages, which are also reviewed in the present article, may also be superior from the public health perspective and decrease the risk of zoonoses, including those related to A/H5N1, although there are some hurdles that this technology is yet to overcome.

## 2 Potential benefits of cultured meat

**Figure 1:**
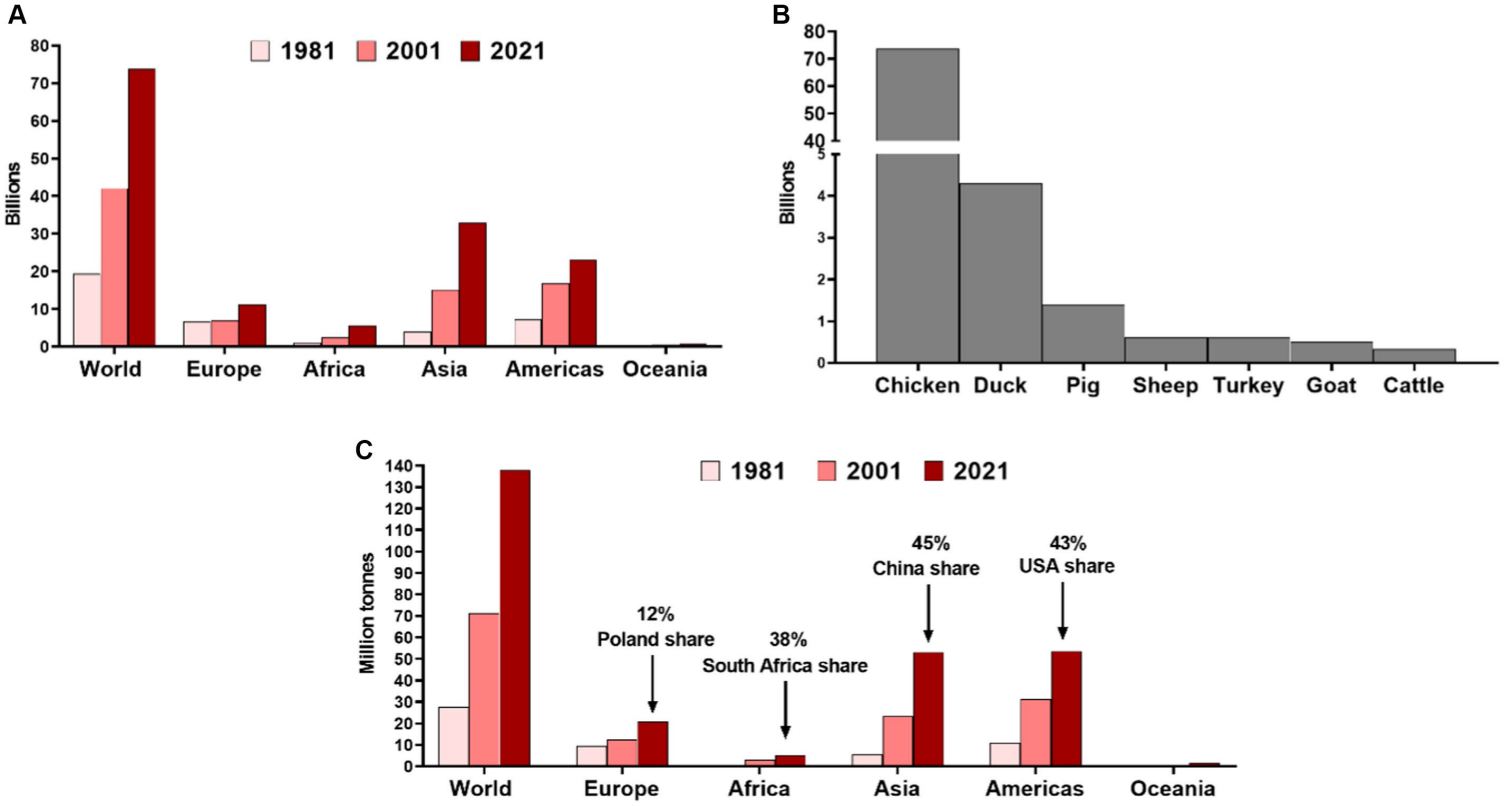
**(A)** Number of chickens slaughtered annually for meat in different world regions in 1981, 2001, and 2021. **(B)** Number of different animals slaughtered globally for meat in 2021. Prepared based on data from the Food and Agriculture Organization of the United Nations collected by Our World in Data (Our World in Data, 2023b). **(C)** The poultry meat production outputs in different world regions with the share of main producers in particular continent (FAOSTAT, 2023).

There are numerous benefits that can arise from the introduction of cultured meat, including cultured poultry. Firstly, it does not require the use of slaughtered animals and, therefore, is ethically superior, especially if one considers that the number of animals slaughtered for meat is increasing (Figure 1). In 2021 alone, 73.8 billion chickens, 1.4 billion pigs, 617 million sheep, 603 turkeys, 501 million goats, and 332 million cattle were slaughtered for meat (Our World in Data, 2023b). The global poultry meat production output amounted to nearly 138 million tonnes in 2021 (Figure 1), with the highest share of China (17.2%), United States (16.8%), Brazil (11.0%), Russia (3.3%), Indonesia (2.8%), India (2.7%), Mexico (2.7%), Poland (1.8%), Japan (1.8%), and Argentina (1.7%) (FAOSTAT, 2023).

Secondly, cultured meat can also offer a number of environmental advantages, including a significant reduction in land use, water withdrawal, energy consumption, and greenhouse emissions (Tuomisto and de Mattos, 2011; Lynch and Pierrehumbert, 2019). For example, the recent analysis based on data collected from over 15 companies and research institutes active in the production and supply chain of cultured meat demonstrated the particular benefits in this regard could be achieved when producing beef and pork with a carbon footprint reduced by over 95 and 9% compared to the global average from conventional production in 2018, respectively (Sinke et al., 2023). In the case of chicken production, the carbon footprint between conventional production and cultured meat does not differ, but the latter is postulated to significantly decrease land use and acidification (Sinke et al., 2023). Using renewable energy sources and optimizing culture medium composition may further reduce environmental impacts (Rzymski et al., 2021; Tuomisto et al., 2022).

Thirdly, the production of cultured meat is a more efficient way of turning crops into meat, regardless of the type of conventional meat it was compared to, including chicken, in case of which an estimated feed conversion rate was two-fold lower (Sinke et al., 2023).

Fourth, the production span of cultured meat, depending on the type of meat, is 2–8 weeks (shorter for chicken, longer for beef) (Bellani et al., 2020; Swartz, 2021) as opposed to 2 months to produce chicken, 6 months for swine, and 2 years for cattle (USDA, 1996, 2018). This makes production more predictable and stable, which is important from the food safety perspective, particularly if one considers the predicted climate change impacts on the food system (Miraglia et al., 2009; Nelson et al., 2014; Sharma et al., 2022).

Fifth, cultured meat has some advantages regarding public health (Halabowski and Rzymski, 2020; Rzymski et al., 2021). These include (i) a decrease in antibiotic use and the contribution of meat production to antibiotic resistance, which is recognized as one of the major human health threats (Antimicrobial Resistance Collaborators, 2022), (ii) better control over bacterial and fungal contamination of the final product, (iii) elimination of macroparasite presence, e.g., *Taenia* sp., *Trichinella* spp., in meat, and (iv) unpredictable emergence and spread of animal viruses, such as African Swine Fever virus or avian influenza viruses that can cause substantial economic damage (Rzymski et al., 2021; Ruiz-Saenz et al., 2022; Farahat et al., 2023). In addition, avian influenza is also a potential threat to human health. As stated in the guidance of the European Center for Disease Prevention and Control (ECDC) released in July 2023: “Transmission to humans cannot be excluded when avian influenza is circulating in wild birds and mammals, and people are directly exposed without wearing protective equipment” (ECDC, 2023b). Over the last 3 years, avian influenza has caused large outbreaks in wild birds and poultry in Europe. In June 2023, the A/H5N1 outbreak in domestic cats was documented in Poland (ECDC, 2023a; General Veterinary Inspectorate, 2023a,b,c). This is particularly concerning since it allows the virus to adapt to mammals, including humans, through reassortment between avian and mammalian influenza viruses (Marschall and Hartmann, 2008). Therefore, any technology that would offer to produce meat but decrease the risk of circulation of avian influenza viruses in poultry and its further spread to mammals, including humans, is highly needed, as discussed in the subsequent sections. One should note that the risk of viral infections, including infections with avian influenza viruses, is not non-existent in the industrial production of cultured poultry. However, it is significantly limited due to no contact with wild birds, sanitation measures, automatization of the process, and the ability to test cells deposited in the master-cell banks for avian pathogens to ensure no introduction of avian influenza viruses (Rzymski et al., 2021; Pasitka et al., 2022). Contrary to this, it is virtually impossible to test every farmed bird individual for avian influenza. The pre-market assessment of cultured poultry meat authorized in the United States revealed that the tested products were free of influenza A (as well as B) viruses (FDA, 2022).

In conclusion, cultured meat, including cultured poultry, is ethically superior, while its introduction to diet is not associated with excluding animal-derived products. It also provides substantial environmental benefits, which, in the case of chicken, particularly involve decreased land use and acidification impacts. Production of cultured poultry is more predictable and less prone to uncontrolled environmental factors, which is important from a food security standpoint. There are also premises that it also offers substantial benefits for public health, including decreased promotion of antibiotic resistance and risk of zoonoses.

## 3 A threat of avian influenza

Avian influenza viruses type A (Orthomyxoviridae family) naturally circulate in various wild birds and can infect poultry (Shriner and Root, 2020; Rafique et al., 2023). These viruses are classified into different subtypes based on hemagglutinin (H) and neuraminidase (N) genes, with 16 H (H1-H16) and 9 N (N1-N9) subtypes of avian influenza identified in birds. Based on clinical significance for poultry, the avian influenza viruses can be classified into low pathogenic and highly pathogenic (HPAI) (Spackman, 2020). The infection with the former is usually mild or asymptomatic, while the latter is characterized by severe disease and high mortality rates (Verhagen et al., 2011). However, when H5 and H7 avian influenza subtypes infect poultry, they may mutate into highly pathogenic forms (Horimoto et al., 1995; Alexander, 2000; Banks et al., 2001; Alexander and Brown, 2009; Monne et al., 2014; Shriner and Root, 2020). With time, the circulation of HPAI has become more frequent, leading to substantial economic loss in the poultry industry. For example, from 1959 to 1995, the emergence of HPAI was documented on 15 occasions with limited losses, but between 1996 and 2008, four outbreaks of HPAI affected millions of birds (Alexander and Brown, 2009). The most recent HPAI outbreak involves A/H5N1, the virus isolated for the first time in 1996 from a sick goose during an outbreak in Guangdong province, China (Xu et al., 1999), which over the years had diverged into distinct lineages and expanded into various reservoir hosts (Sonnberg et al., 2013). In the 2021–2022 epidemic season, a total of 2,467 outbreaks in poultry were identified in Europe alone, with 48 million culled birds. In addition, 187 events of HPAI A/H5N1 were detected in captive birds and 3,573 in wild birds (European Food Safety Authority, European Centre for Disease Prevention and Control, European Union Reference Laboratory for Avian Influenza et al., 2022). The A/H5N1 epidemic continues to cause significant impacts also in both Americas, resulting in 40 million animal losses in the United States in 2022 alone, with economic costs estimated in the range of 2.5–3.0 billion USD (Farahat et al., 2023). The situation continues to evolve in 2023 in various world parts (European Food Safety Authority, European Centre for Disease Prevention and Control, European Union Reference Laboratory for Avian Influenza et al., 2023). One should note that when an A/H5N1 outbreak occurs in poultry, the affected flock is subjected to depopulation, often requiring the culling of healthy birds to prevent further viral spread. This further undermines the ethics of poultry production and highlights that A/H5N1 leads to substantial waste generation, loss of resources, and protein wastage, making conventional poultry production less sustainable (WOAH, 2021).

In general, avian influenza viruses reveal a strong preference for using α2,3-linked sialic acid as a receptor, which dominates in birds. They usually exhibit low infectivity in mammals due to the prevalence of α2,6-linked sialic acid in the respiratory tract (Couceiro et al., 1993; Kimble et al., 2010). In order to become infective for mammals, including humans, several amino acid changes, mostly in the hemagglutinin protein, must occur. They may result from spontaneous mutations, to which influenza viruses are highly prone since their RNA polymerases lack proofreading activity (Boivin et al., 2010). The other process involves the reassortation of genome segments between distinct influenza stains in coinfected cells (Taylor et al., 2023). The first A/H5N1 outbreak virus in humans was documented in Hong Kong in 1997 when 18 individuals were infected by direct contact with chickens, of whom six died (Shortridge et al., 1998). As of 31 May 2023, 876 human infections with A/H5N1 were reported to the World Health Organization between 2003 and 2023, with a high mortality rate of 52% (WHO, 2023a). Only five cases were noted during the recent A/H5N1 epidemic in Europe (2021–2023), with no deaths reported (WHO, 2023a). Moreover, two of these cases that occurred in poultry workers in Spain are suspected not to represent real viral infections but rather arise from environmental contamination of nasal mucosa (Aznar et al., 2023). At the same time, a rising number of terrestrial and aquatic mammalian species have been identified to be infected with this virus, resulting in mortality and causing concern about the potential threat to public health. These species mostly include wild animals or those kept in captivity, e.g., in zoos (Keawcharoen et al., 2004; Kaplan and Webby, 2013). No transmission between these animals and humans has been evidenced in the past. However, recent investigations documented A/H5N1 transmission in minks farmed in 2022 in Spain (Agüero et al., 2023). It is also suspected that it may spread between seals in coastal New England in 2022 (Puryear et al., 2022).

In summary, A/H5N1 is a significant threat to food supply and security, requires culling a high number of birds, including healthy individuals, only to contain outbreaks, leads to substantial resources and economic losses, and continues to create a risk to human health. These risks are especially concerning given the increased outbreaks of A/H5N1 in mammals, including those with close contact with humans, such as domestic cats (WHO, 2023c).

## 4 A/H5N1 infections in cats

The first cases of A/H5N1 infections in felids were noted in 2003, and within the last two decades, nearly 10 species belonging to the Felidae family have been affected, including wild and domestic cats (Table 1). These findings demonstrate that A/H5N1 can eventually evolve into genotypes posing a threat to mammals, and this risk cannot be neglected, given the devastating outcomes such infections could have on human health (Abbasi, 2023). The risk of the introduction of A/H5N1 to the human population is particularly high when companion animals, such as cats, are affected because of close contact and the tendency of cat owners to increase caregiver burden when a cat becomes ill (Spitznagel et al., 2023). However, A/H5N1 infections in companion animals such as dogs and domestic cats have so far been very sporadic (Songserm et al., 2006a,b).

**Table 1:** History of documented A/H5N1 outbreaks in felids.

Year	Affected species (number of individuals)	Inhabited environment (country)	Suspected A/H5N1 source	Clinical symptoms	Outcome	Reference
2003	*Panthera tigris* (2) *Panthera pardus (2)*	Zoo (Thailand)	Raw chicken meat	High fever, respiratory distress	*Death*	Keawcharoen et al. (2004)
2004	*Panthera tigris*	Zoo (Thailand)	Raw chicken meat; tiger-to-tiger transmission	High fever, respiratory distress, neurological signs	33 deaths 50 euthanized	Thanawongnuwech et al. (2005)
2006	*Felis catus* (2)	Household (Iraq)	Contact with poultry	Not reported	Death	Yingst et al. (2006)
2006	*Felis catus* (3)	Outdoor (Germany)	Unknown	Unknown	Death	Starick et al. (2008)
2006	*Felis catus* (3)	Animal shelter (Austria)	Contact with wild birds	Asymptomatic	Survival	Leschnik et al. (2007)
2009	*Panthera leo (2)* *Panthera tigris (8)* *Catopuma temminckii (2)* *Panthera pardus (3)* *Neofelis nebulosa (1)*	Rescue center (Cambodia)	Raw chicken meat; contact with infected wild birds	Lethargy, anorexia	Survival	Desvaux et al. (2009)
2013	*Panthera tigris* (1)	China (Zoo)	Raw chicken meat	Vomiting, respiratory distress	Death	He et al. (2015)
2014–2015	*Panthera tigris (3)*	China (Zoo)	Raw chicken meat	Vomiting, high fever, respiratory distress	Death	Hu et al. (2016)
2022-	*Lynx rufus* *(2)* *Panthera tigris* (1) *Panthera pardus* (1)	USA	Unknown	Respiratory distress	Euthanized	Harvey et al. (2023) and USDA (2023a)
2022	*Felis catus (1)*	Duck farm (France)	Contact with farmed birds	Apathy, mild hyperthermia, pronounced neurologic signs, dyspnea	Euthanized	Briand et al. (2023)
2023	*Lynx rufus* *(3)* *Puma concolor* (12)	USA	Unknown	Not reported	Death	Harvey et al. (2023) and USDA (2023a)
mid-2023	*Felis catus (1)*	Poultry farm (Italy)	Contact with farmed birds	Not reported	Survival	Della Prevenzione Sanitaria DG (2023)
mid-2023	*Felis catus (3)*	Animal shelters (South Korea)	Commercial cat food	Respiratory distress	Death	Jaeeun (2023) and Korea Times (2023)
2023 (June–July)	*Felis catus (33)* *Caracal caracal (1)*	Outdoor and indoor (Poland)	Raw chicken meat?	Dyspnea, bloody diarrhea, neurological signs	Death or euthanized (in the majority of cases)	General Veterinary Inspectorate (2023d), Rabalski et al. (2023), and WHO (2023b)

Recently, an outbreak of A/H5N1 in domestic cats has been identified in different parts of Poland (WHO, 2023b). The first A/H5N1 cases in cats were confirmed by the General Veterinary Inspectorate in Poland on 26 June 2023, with new cases evidenced in subsequent days – the involved cats included household and free-ranging individuals, which died after experiencing fever, dyspnea, and neurological symptoms (General Veterinary Inspectorate, 2023a,b,c). These symptoms are in line with previous A/H5N1 infections documented in felids (Keawcharoen et al., 2004; Marschall and Hartmann, 2008; Thiry et al., 2009; Harder and Vahlenkamp, 2010). On 30 July 2023, the first genomic sequence of A/H5N1 isolated from the oro-pharyngeal swab of a dead cat was deposited in GISAID (ID: EPI_ISL_17949824). Genomic analysis of the virus sequences obtained from other dead cats from various locations in Poland indicated that they all belonged to the H5 clade 2.3.4.4b and were highly related to each other, suggesting a common origin (e.g., ID: EPI_ISL_17951056, EPI_ISL_17951055, EPI_ISL_17951054, EPI_ISL_17951053, EPI_ISL_17951052, EPI_ISL_17951051, and EPI_ISL_17950995) (WHO, 2023b). In addition, the isolates revealed two mutations in the gene encoding PB2 protein, i.e., E672K and K526R, that have been previously recognized to expand the host range of A/H5N1 and adapt it better to replication in mammalian cells (Long et al., 2013; Song et al., 2014; Kupferschmidt, 2023). The phylogenetic trees generated for each genome segment of HPAI H5N1 viruses from cats and avian species in Poland and H5 sequences collected in different European countries are available in the work by Domańska-Blicharz et al. (2023). Their topology indicates that the HPAI H5N1 viruses collected from the cats belonged to the CH (H5N1_A/Eurasian_Wigeon/Netherlands/3/2022-like) genotype (Domańska-Blicharz et al., 2023). According to the World Health Organization, as of 11 July 2023, 47 samples from 46 cats and one captive caracal have been tested for A/H5N1, of which 29 (61.7%) were positive. At least 23 of the cats were indoor individuals, with 18 having access to a balcony, backyard, or terrace and 5 having no access to the outside environment. As of 30 June 2023, 11 infected cats were reported to die, and 14 were euthanized. As noted, this outbreak is the first to report a high number of infected cats over a wide geographical area within a country (WHO, 2023b). By mid-July 2023, samples obtained from 33 domestic cats and one caracal were confirmed to be A/H5N1-positive (General Veterinary Inspectorate, 2023d) One should also note that a real morbidity and mortality was likely higher due to underreporting.

It has been recognized for a long time that an outbreak of A/H5N1 infections in domestic cats, particularly household, may create the opportunity for a virus to adapt to a mammalian host and potentially spread in humans (Songserm et al., 2006b; Marschall and Hartmann, 2008). This is particularly a threat if cases of feline A/H5N1 infection coincide with increased human influenza cases, which in the temperate zone are noted between autumn and early spring (Marschall and Hartmann, 2008; Tamerius et al., 2011). Although the route of infection of A/H5N1 in cats in this particular outbreak remains to be elucidated, it is certainly plausible that infections with avian influenza viruses in domestic cats may occur through the consumption of bird meat, including raw chicken, which is often offered to them by owners (Thiry et al., 2009). This hypothesis is supported by (i) the genomic similarities of A/H5N1 isolated from different cats, (ii) numerous cases of avian disease over a short period of time, which occurred in domestic cats, including indoor individuals, from different locations in Poland, often hundreds of kilometers apart, (iii) the fact that infections occurred during the breeding season for wild birds, which are then characterized by limited migration, and (iv) similarity of viral sequences to those of H5 clade 2.3.4.4b viruses causing an outbreak in poultry in Poland (although they were also similar to those circulating in wild birds; WHO, 2023b). In line with this, the World Organization for Animal Health/Food and Agriculture Organization Network of Expertise on Animal Influenza stated that a direct role from infected wild birds is unlikely as a common source of A/H5N1 in this particular case since not all infected cats had outdoor access, while the wide geographical distribution of infections in cats excludes cat-to-cat transmission as a primary mode of spread (WOAH/FAO, 2023).

The investigation of five frozen meat samples provided by caregivers of cats infected by A/H5N1 in Poland confirmed high levels of viral RNA in a sample of chicken meat purchased fresh and intended for human consumption and which was consumed by the household, A/H5N1-infected cat prior to the emergence of symptoms (Rabalski et al., 2023). Considering that viral sequences isolated from different cat-derived samples were nearly identical, although infected animals inhabited different geographical locations in the country, it is most plausible that they shared the same infection source (Rabalski et al., 2023). Notably, the cat-derived sequences were also highly similar to sequences of A/H5N1 isolated from chicken meat (bearing E627K and K526R mutations). In addition, an A/H5N1 virus isolated from the chicken meat demonstrated the ability to infect both canine and feline cells *in vitro* and was found to infect human airway epithelia in cell culture and induce cytopathic alterations (Rabalski et al., 2023).

Importantly, feed based on raw chicken was already suspected as a source of A/H5N1 infection in other outbreaks in felids (Table 1), and such a route of transmission in *Felis catus*, has also been evidenced experimentally (Kuiken et al., 2004; Rimmelzwaan et al., 2006). Repeatedly reported cases of A/H5N1 infection in domestic cats from different parts of the world, including cases in which feed based on raw chicken or contact with farmed birds was the most probable source of the virus, should be regarded as a warning sign and motivate the development of solutions that could mitigate future transmissions and reduce the risk of A/H5N1 adaptation to a human host through an intermediate host, such as domestic cat or other company animals that come in close contact with humans. To this end, the subsequent section discusses cultured poultry meat technology as one of many potential measures of mitigation strategy worth being considered.

## 5 Is slaughter-free poultry a solution?

The present situation of the A/H5N1 epidemic, increasing economic loss, and potential health threats indicate the urgent need to implement various mitigation strategies, including active surveillance, biosecurity measures, culling of affected poultry, and vaccination (Bertran et al., 2018; Hill et al., 2018). Nevertheless, due to the increasing number of poultry farmed in different world regions, the potential reservoir of A/H5N1 to emerge in poultry and spread to other birds and animals is expanding. Between 2000 and 2021, the number of chickens slaughtered globally for meat increased by over 33 billion and nearly doubled in Europe (Figure 1). Poultry meat production is expected to increase at a 1.9% annual rate (Alkhtib et al., 2023; Our World in Data, 2023a). Therefore, this clearly creates the need to find alternative approaches to meet the demand and decrease various risks arising from poultry production, including those related to A/H5N1.

This is also important if one considers that the HPAI viruses were demonstrated experimentally to survive up to several days on raw poultry meat at varying temperatures (Dai et al., 2022). There are some reports on the presence of such viruses in duck and chicken meat (Wu et al., 2020; Rabalski et al., 2023). This ability increases the risk of their transmission and infection, e.g., during contact with meat products and their further processing. Moreover, in some flocks, A/H5N1 infections may not be accompanied by increased mortality or clinical signs of disease and ultimately remain undetected (Gobbo et al., 2022). In addition, the HPAI viruses, including A/H5N1, can also survive on tissues and feathers of bird carcasses for some time in infective form, creating the risk of transmission when handling culled individuals due to avian influenza outbreaks on farms (Yamamoto et al., 2017). There are some reports from developed countries on human infections with A/H5N1 in individuals involved in culling birds in response to viral outbreaks (Adlhoch et al., 2023). Last but not least, biosecurity measures are often not effective enough in controlling avian influenza even in developed countries as highlighted by nearly 2,500 outbreaks of A/H5N1 reported in European poultry between 2021 and 2022, resulting in the culling of nearly 48 million domestic birds in 37 countries (European Food Safety Authority, European Centre for Disease Prevention and Control, European Union Reference Laboratory for Avian Influenza et al., 2022). All in all, there is an urgent necessity to introduce changes to poultry meat production to limit the economic loss and decrease health risks associated with avian influenza. Although, at present, cultured meat does not offer a viable solution to these issues, it may potentially decrease the avian influenza threats in the future if the technology undergoes further development, scaling up, and introduction to different markets.

With the recent United States Department of Agriculture approval of cultured poultry produced by two companies, more producers are expected to apply for evaluation in the United States (FDA, 2022, 2023). In late 2020, cell-cultured chicken products (containing 70% cultured chicken cells hybridized with plant proteins) were also authorized in Singapore (Singapore Food Agency, 2020). As of September 2023, no other country authorized such products, but there has been substantial interest and investments in their development in Israel, Netherlands, United Kingdom, China, South Korea, Japan, France, and Spain (Food Industry Executive, 2023). In May 2023, the European Food Safety Authority reported that it is ready to pursue a scientific assessment of cultured meat products (European Food Safety Authority, 2023).

Their introduction may provide various benefits, as already discussed in the previous section of this article, and which also encompass health issues (Table 2). It will likely decrease foodborne illnesses, which is substantial for poultry and accounts for the highest number of food-related hospitalizations in the United States (Chai et al., 2017). However, it should also be regarded as a chance to limit the economic losses and health threats associated with A/H5N1 circulation. Although all these benefits could be achieved by switching to plant-based diets (Rzymski et al., 2021; Gibbs and Cappuccio, 2022; Pieczyńska and Rzymski, 2022; Carey et al., 2023), they cannot be regarded as the ultimate solution. A relevant percentage of the human population is unwilling to exclude or limit meat from their diet while developing plant-based alternatives that sufficiently mimic the organoleptic and nutritional parameters of livestock products is challenging, and there are additional barriers related to cost and allergenicity issues (Graça et al., 2015; Verhoeckx et al., 2016). Cultured meat offers an alternative that does not force a consumer to abandon the products he/she is attached to. At the same, the production of cultured poultry does not involve continuous animal farming and decreases the risk of A/H5N1 mutation and cross-species infections. Furthermore, the production of cultured poultry allows one to produce raw meat for companion animals, including cats, which are obligatory carnivores, should not be fed plant-based products, and are frequently given raw meat, including poultry, by their caregivers. Using cultured meat in the diet of household cats creates a much lower risk of avian influenza transmission and, in addition, may decrease the ecological footprint of these animals, which was calculated to constitute 25% of the environmental impact of meat consumption in the United States (Okin, 2017).

**Table 2:** The main advantages and challenges of poultry obtained with cultured meat technology as compared to conventional meat production.

Dimension	Advantages	Challenges
Health	Decreased risk of zoonoses Decreased use of antibiotics and reduced impacts on antibiotic resistance promotion	Unrecognized
Environment	Lower ecological footprint due to decreased land use and acidification	Optimization of energy use and water recycle
Production	Better food security	Matching the taste, texture, and color of different varieties of conventional poultry meat Scaling the production to a level competitive with the conventional one, meeting the demand for reagents (particularly culture media), and building novel production facilities
Public perception	Ethical superiority	The price of the final product Public acceptance

This said, one should note that cultured meat production is also met with several challenges (Table 2). To be fully ethically superior, the production of cultured meat cannot involve animal serum (e.g., fetal bovine serum), which is classically used in *in vitro* cell cultures to support their growth. Instead, non-animal substitutes should be employed. The efforts to develop and introduce them are being pursued, with some animal-origin-free media already commercialized, but it is yet to be shown whether they perform similarly to animal serum in culturing cells for meat production while being economically affordable (Benjaminson et al., 2002; Gstraunthaler, 2003; Piletz et al., 2018; Rzymski et al., 2021; Yamanaka et al., 2023). Nevertheless, there has apparently been progress in producing cultured chicken meat without animal serum. As long as the first products of this type authorized in Singapore in 2020 were produced using small quantities of fetal bovine serum, in early 2023, the approval for cultured chicken meat produced using serum-free media was granted. According to the documents submitted by the manufacturer to the Food and Drug Administration, cultured meat of *Gallus gallus* can be produced with and without serum media (FDA, 2022), although the company states to phase out the use of bovine serum albumin with recombinant forms of albumin protein (Food Navigator USA, 2022).

Moreover, a significant obstacle in introducing cultured poultry obtained with cultured meat technology may be its price, although it can only be projected at this point. According to one analysis, the wholesale cost of cell-cultured meat obtained using large-scale production was optimistically estimated at as high as 63 USD per kg (Garrison et al., 2022). However, CE Delft’s TEA study has projected that reducing the costs of growth factors and limiting the use of recombinant proteins (e.g., albumin, insulin, transferrin) could reduce the production costs to 15 USD per kg, still exceeding those of conventional products (Vergeer et al., 2021). Further reductions in the requirement for payback time, production run time, and volume of cultivated cells could eventually put down the costs to approx. 6 USD per kg (Vergeer et al., 2021), below the price of chicken filets on the US market and the majority of European ones (Numbeo, 2023). However, when these targets could be reached remains unknown. In order to fulfill consumers’ expectations and gain acceptance, cultured poultry must also match (or be superior to) the taste, structure, texture, flavor, nutritional quality, and overall appearance of the conventional meat counterpart (Ye et al., 2022). The difficulty of reaching these goals is likely higher for some meat types, e.g., beef steaks or pork chops (Fraeye et al., 2020). Whether cell-based poultry meat can be comparable to its conventional counterpart requires comparative consumer studies, preferentially blinded, the results of which are not available at the moment. According to the data presented to the U.S. Food and Drug Administration over the course of the first pre-marketing assessment, compared to standard counterparts, serum-free cultured chicken revealed lower levels of total fat, saturated fat, monounsaturated and polyunsaturated fatty acids, and sodium, similar contents of moisture, total amino acids, niacin (B3), magnesium and manganese, but higher levels of cholesterol, pantothenic acid (B5), pyridoxine (B6), vitamin A, calcium, iron, potassium, phosphorus, selenium, and zinc (FDA, 2022). Although some of these differences could be perceived as a nutritional advantage, further studies are needed to understand their effect on the general consumer’s reception of the product. Importantly, serum-free chicken contained higher levels of lead and cadmium compared to conventional ground chicken (FDA, 2022). It is also important to decrease the carbon footprint of cultured chicken production, which was recently shown to be similar to that of conventional products (Sinke et al., 2023). Therefore, it is critical to ensure that the production of cultured meat will rely on renewable energy sources, a factor that may likely also increase the public acceptance of such products. Consumer acceptance itself is also a challenge as cultured meat may appear unnatural, especially in more conservative groups, with lower openness to novelty and high skepticism over scientific achievements (Bryant and Barnett, 2018; Sikora and Rzymski, 2023). It is essential to ensure the appropriate communication of aims, advantages, and shortcomings of cultured meat to provide balanced and fair views to the potential consumers; otherwise, it may be subject to similar opposition as in the case of genetically modified foods (Sikora and Rzymski, 2021). As recently shown, both proponents and opponents of the cultured meat concept raise concerns over safety issues (Sikora and Rzymski, 2023). Last but not least, scaling the production to the level of having a substantial contribution to the global production of poultry meat remains challenging if one considers that poultry meat production in 2021 amounted to 137,979,970.00 tonnes, representing a 93.5% increase compared to 2001 (Figure 1). Some cultured meat facilities in the United States are planned to potentially produce approximately 13,607 tonnes of cultured meat (30 million lbs) a year (UPSIDE Foods, 2023), which would constitute only 0.05% of the poultry meat produced in this country in 2021 (FAOSTAT, 2023). Moreover, switching to cultured poultry technology would require scaling up the production and delivery of various reagents, i.e., culture media (which is the leading cost driver) and growth factors (O’Neill et al., 2022). As calculated, up to 50 L of culture media is required to produce 1 kg of cultured meat (Post, 2014). Therefore, despite the potential benefits that cultured poultry could deliver, it can be expected that unless novel circumstances arise or significant developments in scaling up the manufacturing are made, it may, for a long time, play only an accessory role in the global production output. Despite this, it may still decrease the pressures and threats of transmitting highly pathogenic avian influenza in some world regions.

## 6 Conclusion

The spread of the HPAI A/H5N1 virus in poultry leads to significant economic loss and concerns over the emergence of avian influenza in humans. It leads to a continuous need to pursue various mitigation strategies and seek novel solutions to the problem. This paper postulates that introducing cultured poultry meat is ethically superior, has environmental advantages, provides various public health benefits, and could also be regarded as a potential opportunity to mitigate the impacts and threats of avian influenza. By shifting poultry production to the cultured meat industry, the frequency of A/H5N1 outbreaks in farmed birds may decrease, leading to a reduced risk of virus acquisition by wild and domesticated mammals (including company animals, such as cats and dogs) that have direct contact with birds or eat raw poultry, ultimately minimizing the potential of A/H5N1 to adapt better to mammalian host, including human. This strongly advocates the further development and implementation of this technology in the context of poultry meat, even though, at present, it may be challenging to scale it up to the level competitive to conventional production.

## Author contributions

PR: Conceptualization, Investigation, Methodology, Project administration, Visualization, Writing – original draft, Writing – review & editing.
